# Surface Pretreatment Protocols For Indirect/Semi-Direct Dental Restorations: A Cross-Sectional Survey and Expert Consensus

**DOI:** 10.3290/j.jad.c_2106

**Published:** 2025-06-19

**Authors:** Felicitas Mayinger, Valerie Lankes, Malgorzata Roos, Nadja Rohr, Alexis Ioannidis, Adham Elsayed, Jan-Frederik Güth, Daniel Edelhoff, Nicole Passia, Iman Esmail, Florian Beuer, Stefan Wolfart, Benedikt Christopher Spies, Martin Schimmel, Samir Abou-Ayash, Sebastian Hahnel, Maximiliane Amelie Schlenz, Roland Frankenberger, Uwe Blunck, Dominik Kraus, Marcus Engelschalk, Fabian Huettig, Matthias Kern, Anne-Katrin Luehrs, Petra C. Gierthmuehlen, Bogna Stawarczyk

**Affiliations:** a Felicitas Mayinger Dentist, Department of Prosthetic Dentistry, University Hospital, LMU Munich, Goethestraße 70, 80336 Munich, Germany. Edited the survey, collected data, statistical analysis, reviewed the survey finding (expert), wrote the manuscript.; b Valerie Lankes Dentist, Department of Prosthetic Dentistry, University Hospital, LMU Munich, Goethestraße 70, 80336 Munich, Germany. Proposal, Design of the survey, collected data, wrote the manuscript.; c Malgorzata Roos Senior statistician, Department of Biostatistics, Epidemiology, Biostatistics and Prevention Institute, University of Zurich, Hirschengraben 84, 8001 Zurich, Switzerland. Statistical analysis, edited the manuscript.; d Nadja Rohr Dentist, Biomaterials and Technology, Department Research, University Center for Dental Medicine Basel UZB, University of Basel, Mattenstrasse 40, 4058 Basel, Switzerland. Reviewed the survey finding (expert), edited the manuscript.; e Alexis Ioannidis Dentist, Clinic of Reconstructive Dentistry, Center for Dental Medicine, University of Zurich, Plattenstrasse 11, 8032 Zurich, Switzerland. Reviewed the survey finding (expert), edited the manuscript.; f Adham Elsayed Dentist, Department of Prosthetic Dentistry, University Hospital, LMU Munich, Goethestraße 70, 80336 Munich, Germany. Dentist, Department of Prosthodontics, University Hospital Schleswig-Holstein, Campus Kiel, Christian-Albrecht University of Kiel, Arnold-Heller-Straße 3, 24105 Kiel, Germany. Reviewed the survey finding (expert), edited the manuscript.; g Jan-Frederik Güth Dentist, Poliklinik für Zahnärztliche Prothetik, Center for Dentistry and Oral Medicine (Carolinum), Goethe-University, 60596 Frankfurt am Main, Germany. Reviewed the survey finding (expert), edited the manuscript.; h Daniel Edelhoff Dentist, Department of Prosthetic Dentistry, University Hospital, LMU Munich, Goe-thestraße 70, 80336 Munich, Germany. Reviewed the survey finding (expert), edited the manuscript.; i Nicole Passia Dentist, Department of Prosthodontics, Faculty of Medicine and University Hospital Carl Gustav Carus, Technische Universität Dresden, Fiedlerstraße 25, 01307 Dresden, Germany. Reviewed the survey finding (expert), edited the manuscript.; j Iman Esmail Dentist, Department of Prosthetic Dentistry, University Hospital, LMU Munich, Goe-thestraße 70, 80336 Munich, Germany. Design of the survey, edited the manuscript.; k Florian Beuer Dentist, Department of Prosthodontics, Geriatric Dentistry and Craniomandibular Disorders, Charité – Universitätsmedizin Berlin, Aßmannshauser Straße 4-6, 14197 Berlin, Germany. Reviewed the survey finding (expert), edited the manuscript.; l Stefan Wolfart Dentist, Department of Prosthodontics and Biomaterials, Center for Implantology, Medical Faculty, RWTH Aachen University, Pauwelsstraße 30, 52074 Aachen, Germany. Reviewed the survey finding (expert), edited the manuscript.; m Benedikt Christopher Spies Dentist, Department of Prosthetic Dentistry, Faculty of Medicine, Center for Dental Medicine, Medical Center, University of Freiburg, Hugstetter Straße 55, 79106 Freiburg, Germany. Reviewed the survey finding (expert), edited the manuscript.; n Martin Schimmel Dentist, Department of Reconstructive Dentistry and Gerodontology, School of Dental Medicine, University of Bern, Freiburgstrasse 7, 3010 Bern, Switzerland. Reviewed the survey finding (expert), edited the manuscript.; o Samir Abou-Ayash Dentist, Department of Prosthetic Dentistry and Material Science, University Medical Center of the Johannes Gutenberg University Mainz, Langenbeckstraße 1, 55131 Mainz, Germany. Reviewed the survey finding (expert), edited the manuscript.; p Sebastian Hahnel Dentist, Department of Prosthetic Dentistry, University Hospital Regensburg, Franz-Josef-Strauß-Allee 11, 93053 Regensburg, Germany. Reviewed the survey finding (expert), edited the manuscript.; q Maximiliane Amelie Schlenz Dentist, Department of Prosthodontics, University Hospital Schleswig-Holstein, Campus Kiel, Christian-Albrecht University of Kiel, Arnold-Heller-Straße 3, 24105 Kiel, Germany. Reviewed the survey finding (expert), edited the manuscript.; r Roland Frankenberger Dentist, Department of Operative Dentistry, Endodontics, and Pediatric Dentistry, Medical Center for Dentistry, University Medical Center Giessen and Marburg, Georg-Voigt-Straße 3, 35033 Marburg, Germany. Reviewed the survey finding (expert), edited the manuscript.; s Uwe Blunck Dentist, Department of Operative, Preventive and Pediatric Dentistry, Charité – Universitätsmedizin Berlin, Aßmannshauser Straße 4-6, 14197 Berlin, Germany. Reviewed the survey finding (expert), edited the manuscript.; t Dominik Kraus Dentist, Department of Prosthodontics, Preclinical Education and Dental Materials Science, University of Bonn, Welschnonnenstraße 17, 53111 Bonn, Germany. Reviewed the survey finding (expert), edited the manuscript.; u Marcus Engelschalk Dentist, Department of Oral and Maxillofacial Surgery, University Medical Center Hamburg-Eppendorf, Martinistrasse 52, 20246 Hamburg, Germany. Reviewed the survey finding (expert), edited the manuscript.; v Fabian Huettig Dentist, Department of Prosthodontics, Center for Dentistry, Oral Medicine and Maxillofacial Surgery, University Hospital Tuebingen, Osianderstraße 2-8, 72076 Tübingen, Germany. Reviewed the survey finding (expert), edited the manuscript.; w Matthias Kern Dentist, Department of Prosthodontics, University Hospital Schleswig-Holstein, Campus Kiel, Christian-Albrecht University of Kiel, Arnold-Heller-Straße 3, 24105 Kiel, Germany. Reviewed the survey finding (expert), edited the manuscript.; x Anne-Katrin Luehrs Dentist, Hannover Medical School, Department of Conservative Dentistry, Periodontology and Preventive Dentistry, Carl-Neuberg-Straße 1, Hannover 30625, Germany. Reviewed the survey finding (expert), edited the manuscript.; y Petra C. Gierthmuehlen Dentist, Department of Prosthodontics, Medical Faculty and University Hospital Düsseldorf, Heinrich-Heine-University, Moorenstraße 5, 40225 Düsseldorf, Germany. Reviewed the survey finding (expert), edited the manuscript.; z Bogna Stawarczyk Material scientist, Department of Prosthetic Dentistry, University Hospital, LMU Munich, Goethestraße 70, 80336 Munich, Germany. Proposal, design of the survey, statistical analysis, collected data, reviewed the survey finding (expert), wrote the manuscript.

**Keywords:** adhesive dentistry, airborne particle abrasion, parameter, surface conditioning, bonding, dental restoration

## Abstract

**Purpose:**

To investigate, via questionnaire, how protocols for adhesive luting workflows of dental restorations are applied in three German-speaking countries.

**Material and Methods:**

A 47-item questionnaire gathered data on airborne particle abrasion (APA) unit characteristics, parameters, operating procedures, pretreatments in adhesive luting workflows for restorations, and participant demographics. The survey was distributed via trade journals, expert associations, universities, technical schools, and social media. Marginal absolute and relative frequencies were analyzed (95% confidence intervals), with Chi-squared tests comparing observed and expected frequencies (P<0.05). Twenty-three experts voted on 23 recommendations regarding APA parameters and other pretreatments for bonding restorations.

**Results:**

A total of 267 participants completed the survey. Access to an APA unit was linked to a higher likelihood of performing APA before placement. Approximately half of the participants used APA in their practice. For zirconia restorations, 47.2% applied alumina APA at 50 µm/0.1 MPa, while 36.7% used the same settings for polymer-based restorations. For alloys, 37.5% employed 110 µm/0.2 MPa. These preferences correlated with age (≥30 years), experience (≥10 years), profession (dental technician/dentist), prior instruction/training, and daily APA use. Adhesives with MDP were used for zirconia (63.8%) and those with silane for silicate-based ceramics (55.9%). Agreement on recommendations ranged between 52% and 100%, with 21/23 reaching an average of 93%.

**Conclusion:**

Access to APA influenced clinical decisions and the feasibility of adhesive luting workflows. Adequate APA equipment in dental facilities is essential for quality care. Standardized protocols, training, and education across dental professions are necessary to enhance understanding and proper use of APA.

A variety of dental materials undergo airborne particle abrasion (APA), conducted by dental technicians, dentists, and dental assistants. Most APA units allow dental professionals to set various parameters, such as adjusting the pressure up to 1.2 MPa, and to vary the abrasion agent in terms of type and particle size. Users can furthermore alter the abrasion duration, the distance, and the angle between the nozzle and restoration. Among the available APA agents, alumina particles are particularly popular and come in sizes ranging from 25 µm to 250 µm. Depending on the particle size and applied pressure, alumina air abrasion alters the substrate’s surface topography, roughness profile, and surface tension.^37,54^ Beyond cleaning, the surface area is enlarged and the wettability of adhesives or luting materials is increased by the creation of micro-structures, thus improving retention and creating an adequate bond strength.^43^ Tribochemical coating represents another APA technique, offering both surface topography modification and chemical alteration. During APA with silicon oxide-coated alumina particles (CoJet, Rocatec), silicon oxides are propelled onto the substrate surface. These localized deposits of silica-coated alumina are created on parts of the substrate, thereby introducing silica sites that subsequently bond to silane and enhance the overall bond strength.^51^ Glass pearls (sometimes referred to as “glass beads”) and nutshell abrasives are less common in general dental parlance but are used in certain abrasion applications to achieves very gentle surface modification or cleaning. Glass pearls (or beads), generally made from soda-lime or borosilicate glass in sizes from about 25 to 250 µm, have a gentler effect due to their spherical shape and are primarily used for delicate cleaning or finishing, removing only light debris without creating deep roughness. Nutshell abrasives, often walnut shells, come in similar particle sizes and likewise offer a mild abrading action suited to removing investment material or polishing polymer surfaces and dentures without causing substrate damage. Both media are softer than alumina, so they tend to be used for specialized, minimally invasive tasks rather than creating substantial roughness for enhanced bonding.

Zirconia, alloys, polyetheretherketone (PEEK), polymethylmethacrylate (PMMA), and indirect/semi-direct resin composites should be airborne-particle abraded prior to fixation to increase bond strength.^28,30,37,44,46,47,49,55^ It is essential to adapt the APA parameters, especially the pressure and the agent itself, to each material, as improper APA may compromise the flexural strength of the restorative material^91^ due to microcracks and damage to the surface.^90^ In addition, the risk of microleakage and plaque accumulation due to surface roughness in the marginal area of the restoration may be increased,^65^ potentially resulting in clinical failures.

For the adhesive luting of silicate-based ceramics, surface area enlargement is typically achieved by etching with 5–9% hydrofluoric acid.^83^ As hydrofluoric acid etching does, however, have noxious and irritating effects on the organism, it must be handled with extreme caution.^57^ For intraoral repair, buffered compositions have been developed.^1^ Previous investigations have indicated that alumina APA of the bonding surface of silicate-based ceramic restorations can result in similar bonding effectiveness (8–23 MPa) as etching with 5–10% hydrofluoric acid (7–24 MPa).^59,67,88^ However, the biaxial flexural strength after bonding was decreased (134–147 MPa) compared to the pretreatment with hydrofluoric acid (146–154 MPa).^70^

In clinical practice, the bonding area of the restoration is commonly airborne-particle abraded by the dental technician before the restoration is delivered to the dentist. However, during try-in before insertion, contamination with saliva and/or blood often occurs. This contamination can reduce the adhesive strength of the restoration.^53^ Thus, either APA after try-in and/or a subsequent cleaning of the restoration by etching with phosphoric acid or by specially produced cleaning agents (eg, Ivoclean, Katana Cleaner) is recommended before adhesive luting, with the different cleaning protocols showing varying levels of success depending on the restoration material.^8,20^

In addition to a pretreatment with APA and/or hydrofluoric acid etching, the chemical composition of the adhesive system and/or luting material is equally important for the long-term success of the dental restoration. Adhesives can be applied following phosphoric acid etching on both enamel and dentin (“etch and rinse technique”), solely enamel (“selective etch technique”), and/or by using adhesives with self-etch properties. Additionally, new universal adhesives are available, allowing for the pre-treatment of the tooth substrate as well as restorations. Scientific results indicate that with universal adhesives containing 10-MDP (10-methacryloyloxydecyl dihydrogen phosphate) monomer,^82,86^ high bond strength to zirconia is achieved.^4,41,89^ For silicate-based ceramics, the latest investigations also report comparable bond strength values *in vitro* for universal adhesives in comparison with conventional adhesives containing a monosilane.^6,78^

As current practices on the pretreatment of dental restorations seem to vary widely among dental professionals, it was the aim of this study to conduct a survey in three German-speaking countries investigating the clinical use of APA and pretreatments for adhesive placement of dental restorations. The tested scientific hypotheses stated that neither the participants’ sex, age, experience, activity, profession nor their previous contact with APA (access to APA unit, prior experience with APA, frequency of APA, instruction/training) have an impact on their use of APA or workflow for bonding dental restorations. Moreover, an expert panel assessed the results of this survey and evaluated recommendations for general and material-specific adhesive luting workflows for dental restorations.

## MATERIALS AND METHODS

### Survey Design and Ethical Approval

The anonymous cross-sectional survey was designed by four experts in the field (FM, VL, IE, BS) and pre-tested with 20 participants. Feedback from this group was collected and used to refine certain questions for improved clarity. The final questionnaire was created via GoogleForms (Google LLC, Mountain View, USA) and consisted of 37 multiple-choice and 10 multiple-response questions (Table 1 and Table 2). The survey followed the ethics of survey research by ensuring a maintenance of confidentiality and anonymity. The Research Ethics Committee of the Faculty of Medicine, LMU Munich previously approved the study (23-0276 KB).

**Table 1:** A questionnaire showing the first four sections consisting of 42 questions

Question	Answer options
**Part 1:** General characteristics of the airborne particle abrasion unit
1. Is there an airborne particle abrasion unit in your laboratory or dental practice?	❒ yes ❒ no
2. How many cartridges for different abrasion particles does your airborne particle abrasion unit have?	❒ 0 ❒ 1 ❒ 2 ❒ 3 ❒ 4
3. What abrasion particles do you airborne particle abrade with? (multiple responses possible)	❒ alumina ❒ glass pearls ❒ zirconia ❒ nutshells
**Part 2:** General and material-specific airborne particle abrasion parameters
4. Have you previously performed alumina airborne particle abrasion in your daily work?	❒ yes ❒ no
5. How often do you perform airborne particle abrasion?	❒ 1–3 times daily ❒ more than 4 times daily ❒ 1–3 times weekly ❒ not applicable
6. Did you have an instruction or training on alumina airborne particle abrasion?	❒ yes ❒ no
7. Which materials are alumina airborne particle abraded in your laboratory or dental practice? (*multiple responses possible*)	❒ silicate-based ceramics ❒ lithium silicate ceramics ❒ zirconia ❒ polymer infiltrated ceramic networks ❒ polymethylmethacrylate-based resin ❒ composites ❒ alloys
8. Do you perform alumina airborne particle abrasion prior to the fixation of a restoration (if this is necessary for the material)?	❒ yes ❒ no
9. If airborne particle abrasion is performed before the fixation, this is conducted by the: (*multiple responses possible*)	❒ dental technicians ❒ dentists ❒ dental assistants
10. How do you prepare the surface that is going to be airborne particle abraded?	❒ with a pencil/felt-tip pen ❒ water steaming ❒ no pretreatment
11. Do dental technicians, dentists or dental assistants in your working environment communicate with each other about airborne particle abrasion?	❒ yes ❒ no
12. Which parameter(s) do you consider when performing alumina airborne particle abrasion? (*multiple responses possible*)	❒ type of abrasion particles ❒ applied pressure ❒ airborne particle abrasion duration ❒ distance between nozzle and restoration ❒ angle between nozzle and restoration ❒ none of these parameters
13. With what parameters do you alumina airborne particle abrade zirconia?	❒ 50 µm and 0.1 MPa ❒ 50 µm and 0.3 MPa ❒ 110 µm and 0.4 MPa ❒ 250 µm and 0.05 MPa ❒ unclear
14. With what parameters do you alumina airborne particle abrade polymer-based resins?	❒ 50 µm and 0.1 MPa ❒ 50 µm and 0.3 MPa ❒ 110 µm and 0.4 MPa ❒ 250 µm and 0.05 MPa ❒ unclear
15. With what parameters do you alumina airborne particle abrade alloys?	❒ 50 µm and 0.05 MPa ❒ 50 µm and 0.3 MPa ❒ 110 µm and 0.2 MPa ❒ 250 µm and 0.05 MPa ❒ unclear
16. What distance do you maintain between the airborne particle abrasion nozzle and the restoration when performing alumina airborne particle abrasion?	❒ 1–5 mm ❒ 5–10 mm ❒ >10 mm ❒ 10 cm
17. What angle do you use between the airborne particle abrasion nozzle and the restoration when performing alumina airborne particle abrasion? (*multiple responses possible*)	❒ approx 45° ❒ approx 90° ❒ is not considered
18. Where do you get specifications for the airborne particle abrasion parameters? (*multiple responses possible*)	❒ manufacturers’ instructions ❒ internal work instructions ❒ internal training ❒ experience ❒ scientific evidence
19. Can you imagine performing airborne particle abrasion on silicate-based ceramics instead of etching them?	❒ yes ❒ no
20. How long do you airborne particle abrade a polymer-based crown?	❒ o< 20 s ❒ > 60 s
21. Which parameter do you material-specifically primarily adjust at the airborne particle abrasion unit?	❒ type of abrasion particles ❒ applied pressure ❒ airborne particle abrasion duration ❒ distance between nozzle and restoration ❒ angle between nozzle and restoration
22. How do you clean the object from remaining abrasion particles after alumina airborne particle abrasion?	❒ in an ultrasonic bath ❒ by compressed air ❒ by water steaming ❒ by disinfection ❒ no additional cleaning
23. Why do you airborne particle abrade a surface? (*multiple responses possible*)	❒ for cleaning ❒ for divesting ❒ for enlarging the surface ❒ for increasing the wettability
24. Airborne particle abrasion is important for: (*multiple responses possible*)	❒ cleaning the surface ❒ improving the wettability ❒ enlarging the surface ❒ increasing the stability of the restoration
**Part 3:** Operating procedures for the airborne particle abrasion unit
25. How do you work with an airborne particle abrasion unit?	❒ sitting ❒ standing
26. Do you use gloves when performing airborne particle abrasion?	❒ yes ❒ no
27. Do you regularly clean your airborne particle abrasion unit?	❒ yes ❒ no
28. How high is your current cleaning effort?	❒ high ❒ moderate ❒ low
29. How do you clean your airborne particle abrasion unit? (*multiple responses possible*)	❒ by suction ❒ by wiping ❒ using disinfectant wipes ❒ no cleaning of the unit
**Part 4:** Pretreatments for placement dental restorations
30. If a zirconia crown is provisionally fixed with an eugenol containing cement, it can subsequently only be:	❒ luted using conventional adhesives ❒ luted using self-adhesive luting composites ❒ luted using universal adhesives ❒ cemented
31. Do you additionally clean a silicate-based ceramic crown etched with hydrofluoric acid with phosphoric acid?	❒ yes ❒ no
32. Do you additionally clean an airborne particle abraded zirconia crown with phosphoric acid?	❒ yes ❒ no
33. Do you use special cleaning products (eg, Ivoclean or KATANA Cleaner) after surface pretreatment?	❒ yes ❒ no
34. Do you use universal adhesives for luting a zirconia restoration?	❒ yes ❒ no ❒ unclear
35. Do you use universal adhesives for luting a silicate-based ceramic restoration?	❒ yes ❒ no ❒ unclear
36. For luting a zirconia restoration, do you use adhesives with	❒ silanes ❒ MDP monomers ❒ unclear
37. For luting a silicate-based ceramic restoration, do you use adhesives with	❒ silanes ❒ MDP monomers ❒ unclear
38. Working with hydrofluoric acid etching	❒ is harmless ❒ is something I would like to avoid
39. What do you do after trying-in the restoration if it has already been alumina airborne particle abraded?	❒ I do not airborne particle abrade again ❒ after trying-in the restoration, I airborne particle abrade again for cleaning
40. What do you do when the restoration has debonded?	❒ I do not airborne particle abrade again ❒ I airborne particle abrade again
41. Has it already happened that the restoration was no longer insertable after performing airborne particle abrasion?	❒ yes ❒ no
42. Would you like more information regarding the airborne particle abrasion process as part of your training, studies or further education?	❒ yes ❒ no
	

**Table 2:** Participants’ characteristics


Gender	female	45.3%
male	52.4%
diverse	2.2%
Age	<30	29.5%
30–39	30.0%
40–49	19.1%
>50	21.3%
Years of experience	<2	12.0%
2–5	22.5%
6–10	13.9%
>10	51.7%
Active in the dental sector	Yes	97.4%
No	2.6%
Current profession (*multiple responses possible*)	other	2.6%
trainee/student	9.7%
dental assistant	6.0%
dental technician	34.8%
dentist	46.8%


### Questionnaire Structure

The questionnaire was divided into five parts:

Part 1 (Questions 1–3): General characteristics of the APA unit.
Part 2 (Questions 4–24): General and material-specific APA parameters.
Part 3 (Questions 25–29): Operating procedures of the APA unit.
Part 4 (Questions 30–42): Pretreatments for bonding dental restorations.
Part 5 (Questions 43–47): Participant demographics (eg, gender, age, professional experience).

Participants had to answer each question in order to proceed and submit the survey, ensuring a complete dataset.

### Survey Administration and Distribution

The online questionnaire, written in German, was administrated from April 5 to July 14, 2023 and distributed via trade journals and their online presences (Quintessence Publishing, ZWP Online, Teamwork Zahnmedizin, Dental dialogue), expert associations, universities and vocational schools and shared over social media (WhatsApp, Facebook, Instagram, LinkedIn) using a QR code in Germany, Austria, and Switzerland.

### Statistical Analysis

All responses (n = 267) were exported to Excel (Microsoft, Redmond, WA, USA) and analyzed using SPSS (IBM SPSS Statistics 27.0, IBM, Armonk, NY, USA).

Discrete explanatory variables included: Access to an APA unit, experience with APA, frequency of APA use, training (instruction) in APA, gender, age, years of experience in the dental field, current activity (eg, technician, dentist, assistant), and profession.

Discrete primary outcome variables comprised: Performing APA prior to restoration fixation, surface preparation approach, APA use for specific materials (zirconia, polymer-based resins, alloys), nozzle distance and duration for APA, parameter adjustments, restoration cleaning procedure, working position, glove use, cleaning of the APA unit, and negative experiences with APA.

For luting-related outcome variables (eg, use of eugenol-containing cement, phosphoric acid, or cleaning products for different materials, re-abrading previously abraded restorations after try-in, and adhesives), only dentists (n = 127) were included in the analysis.

Marginal absolute and relative frequencies were calculated for all discrete variables and supplemented by 95% confidence intervals (CI) using the Wilson method^85^ where appropriate. Associations between discrete variables were tested with the Chi-squared test (significance at P <0.05). For significant Chi-squared results, observed and expected frequencies were compared to assess effect relevance.

### Expert Panel Evaluation

A panel of 23 experts subsequently reviewed the survey findings. Based on these results, 23 recommendations regarding general and material-specific adhesive luting workflows were formulated. Each recommendation was evaluated by the expert panel using a 5-point Likert scale (strongly agree, agree, uncertain, disagree, strongly disagree). The “level of agreement” for any given recommendation was defined as the combined percentage of “strongly agree” and “agree” responses (Table 3).

**Table 3:** Experts’ recommendations

Recommendation	Level of agreement (%)
An airborne particle abrasion unit should be accessible for pretreatment of dental restorations prior to bonding.	100 (23/23)
Users should be instructed/trained on performing alumina airborne particle abrasion. If feasible, airborne particle abrasion should be performed by users that perform airborne particle abrasion daily and possess extensive work experience.	96 (22/23)
Dental technicians, dentists and dental assistants should communicate about airborne particle abrasion.	100 (23/23)
Surfaces that are going to be airborne particle abraded should be marked with a pencil/felt-tip pen. Surfaces that should not be airborne particle abraded should be protected against accidental exposure to airborne particle abrasion to prevent rough surfaces and a potentially increased plaque adhesion.	91 (21/23)
The type of abrasion particle, the applied pressure, the airborne particle abrasion duration, the distance between the nozzle and restoration and the angle between the nozzle and restoration should be considered when using airborne particle abrasion.	100 (23/23)
Zirconia should be alumina airborne particle abraded using 50 µm and 0.1 MPa.	96 (22/23)
Polymer-based restorations should be alumina airborne particle abraded using 50 µm and 0.1 MPa.	87 (20/23)
Alloys should be alumina airborne particle abraded using 110 µm and 0.2 MPa.	91 (21/23)
If feasible, a distance of 5–10 mm should be maintained between the airborne particle abrasion nozzle and the restoration when performing alumina airborne particle abrasion.	100 (23/23)
If feasible, an angle of 45° should be used between the airborne particle abrasion nozzle and the restoration when performing alumina airborne particle abrasion.	87 (20/23)
For polymer-based restorations, airborne particle abrasion duration should be limited (recommendation for a single-unit FDP: <20 s).	96 (22/23)
Specimens should be cleaned in an ultrasonic bath after performing alumina airborne particle abrasion.	100 (23/23)
Surfaces should be airborne particle abraded to divest, clean, enlarge the surface or increase the wettability and thus increase the stability of zirconia, silicate-based, polymer-based or alloy restorations due to an increase in bond strength.	87 (20/23)
Airborne particle abrasion units should be operated when sitting and wearing gloves.	70 (16/23)
Airborne particle abrasion units should be regularly cleaned.	100 (23/23)
After the provisional fixation with an eugenol-containing cement, a zirconia crown should be fixed with a cement.	57 (13/23)
Prior to silanization, a silicate-based ceramic crown etched with hydrofluoric acid should be cleaned with phosphoric acid.	52 (12/23)
An airborne particle abraded zirconia crown should not be cleaned with phosphoric acid.	78 (18/23)
Universal adhesives may represent a less technique sensitive option for successfully luting zirconia and silicate-based ceramic restorations.	78 (18/23)
Adhesives for luting a zirconia restoration should contain MDP monomers.	96 (22/23)
Adhesives for luting a silicate-based ceramic restoration should contain silanes.	91 (21/23)
Airborne particle abrasion should be performed after trying-in. If a restoration has been contaminated after airborne particle abrasion, it should be airborne particle abraded again or treated using specific cleaning products prior to luting.	100 (23/23)
After debonding, previously airborne-particle-abraded restorations should be airborne-particle-abraded again.	100 (23/23)
	

## RESULTS

In total, 267 participants completed the survey. The participants’ characteristics are summarized in Table 2.

### General Characteristics of the Airborne Particle Abrasion Unit

**Fig 1:**
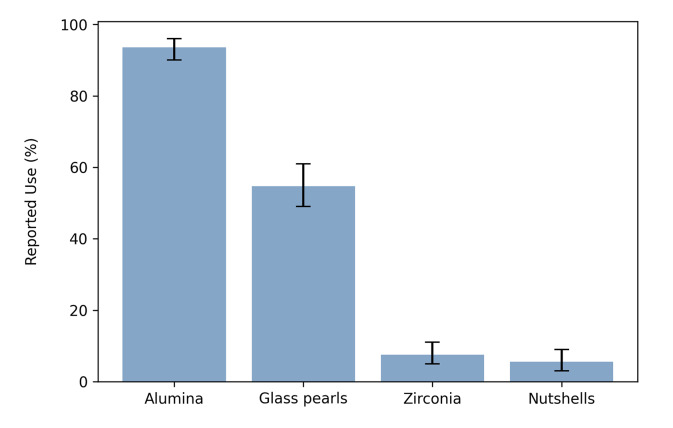
Percentage of abrasive agents used for airborne particle abrasion (with 95% CI).

The majority of the participants (95.9% [95% confidence interval [CI]: 0.93; 0.98]) indicated that an APA unit was part of their laboratory or dental practice. Participants specified the number of cartridges available in their units as follows: 0 (3.7% [CI: 0.02; 0.07]), 1 (10.9% [CI: 0.08; 0.15]), 2 (27.3% [CI: 0.22; 0.33]), 3 (24.0% [CI: 0.19; 0.29]) or 4 (34.1% [CI: 0.29; 0.40]). Regarding the used material, 93.6% [CI: 0.90; 0.96] of the participants used alumina, 54.7% [CI: 0.49; 0.61] glass pearls, 7.5% [CI: 0.05; 0.11] zirconia, and 5.6% [CI: 0.03; 0.09] nutshells (Fig 1).

### General and Material-Specific Airborne Particle Abrasion Parameters

**Fig 2:**
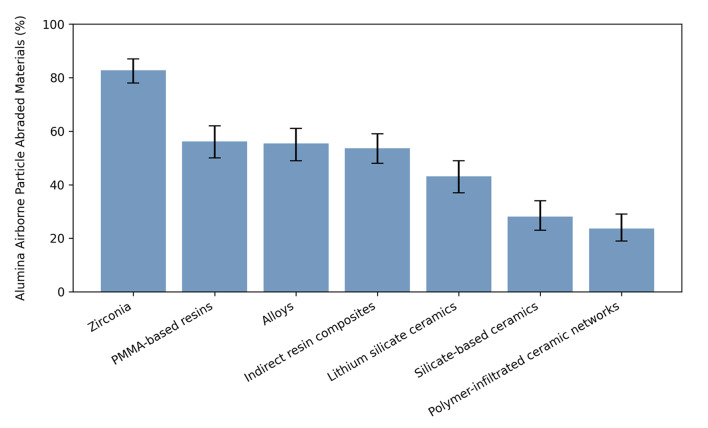
Percentage of use of alumina airborne abrasion for different materials (with 95% CI).

A majority of 86.5% [CI: 0.82; 0.90] reported previous experience with alumina APA. Frequency of use was reported as more than four times per day (24.3% [CI: 0.20; 0.30]), 1–3 times daily (29.6% [CI: 0.24; 0.35]), and 1–3 times per week (32.2% [CI: 0.27; 0.38]). Two-thirds (65.6% [CI: 0.60; 0.71]) of the participants had not received any formal instruction or training on using alumina APA. Most participants (82.8% [CI: 0.78; 0.87]) indicated that they use APA on zirconia. Airborne particle abrasion was applied to polymethylmethacrylate-based resins by 56.2% [CI: 0.50; 0.62], followed by alloys (55.4% [CI: 0.49; 0.61]), indirect/semi-direct resin composites (53.6% [CI: 0.48; 0.59]), lithium-disilicate-glass-ceramics (43.1% [CI: 0.37; 0.49]), silicate-based ceramics (28.1% [CI: 0.23; 0.34]), and polymer infiltrated ceramic networks (23.6% [CI: 0.19; 0.29]) (Fig 2). If necessary for the material, the majority (89.1% [CI: 0.85; 0.92]) likewise performed alumina APA prior to the luting of a restoration.

**Fig 3:**
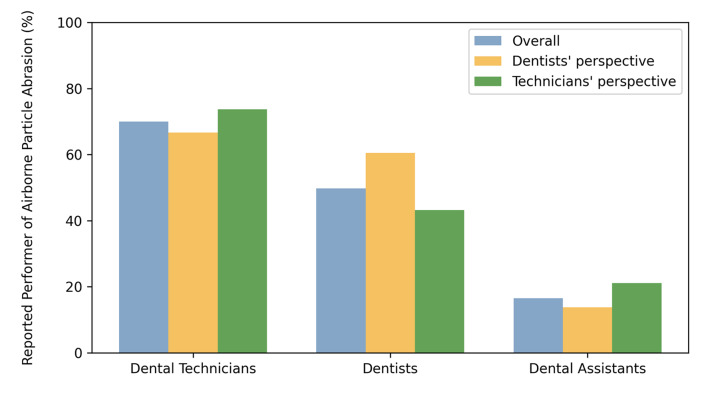
Percentage of primary performer of airborne particle abrasion and differences between the professional groups.

Access to an APA unit was associated with a higher number of participants performing alumina APA prior to luting than was expected (236 vs 228.2; P <0.001). APA was, according to all participants, primarily performed by dental technicians (70% [CI: 0.64; 0.75]), followed by dentists (49.8% [CI: 0.44; 0.56]), and dental assistants (16.5% [CI: 0.13; 0.21]). The analyses of the professional subgroups showed slightly different numbers, with dentists and dental technicians reporting APA to be performed by dental technicians (66.7% vs 73.7%, respectively), followed by dentists (60.5% vs 43.2%), or dental assistants (13.8% vs 21.1%) (Fig 3).

**Fig 4:**
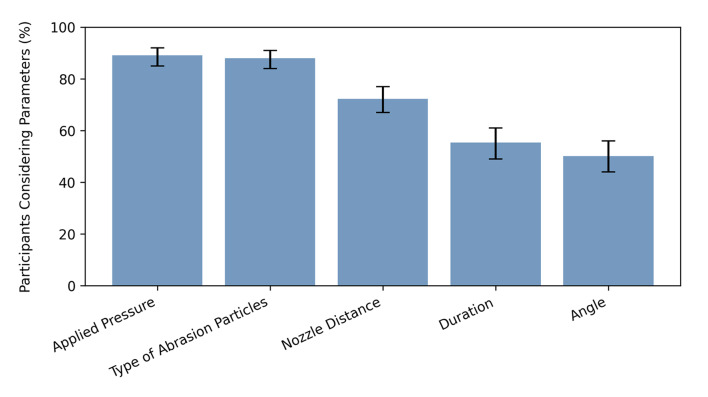
Percentages of considered airborne particle abrasion parameters (with 95% CI).

The majority of the participants (154/267 [CI: 0.52; 0.63]) did not use a pretreatment of the surface prior to APA, while one-third used water steaming (81/267 [CI: 0.25; 0.36]) and 12% (32/267 [CI: 0.09; 0.16]) employed a pencil/felt-tip pen. Just over half of the participants (54.7% [CI: 0.49; 0.61]) reported communicating about APA in their working environment. Key parameters considered by participants included applied pressure (89.1% [CI: 0.85; 0.92]), type of abrasion particles (88.0% [CI: 0.84; 0.91]), nozzle distance to the restoration (72.3% [CI: 0.67; 0.77]), duration (55.4% [CI: 0.49; 0.61]), and angle between nozzle and restoration (50.2% [CI: 0.44; 0.56]) (Fig 4).

**Fig 5:**
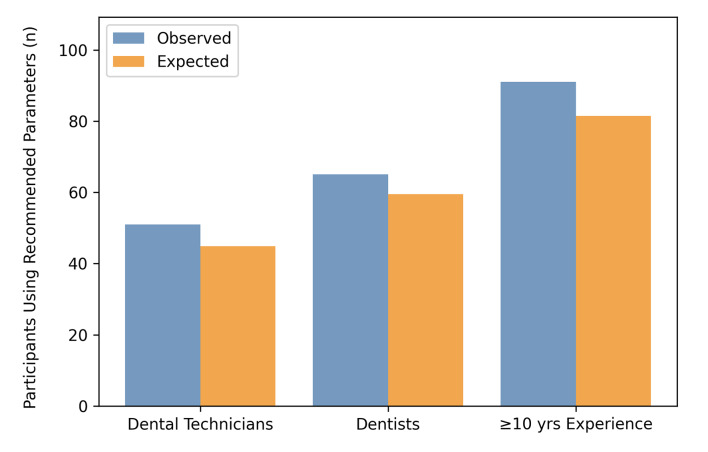
Observed vs. expected adoption of airborne particle abrasion with 50 µm and 0.1 MPa for zirconia (with 95% CI).

Nearly half of the participants (47.2% [CI: 0.41; 0.53]) reported alumina airborne particle abrading zirconia with 50 µm and 0.1 MPa. Being a dental technician or dentist and having 10 years or more of experience were associated with a higher number of participants choosing this surface pretreatment than was expected (51 vs 44.9, 65 vs 59.5, 91 vs 81.5; P = 0.002- 0.44) (Fig 5).

For polymer-based restorations, 36.7% [CI: 0.31; 0.43] used the same settings. An age of 30 or above and an experience of 10 years or more were associated with a higher number of participants employing these parameters for polymer-based restorations (78 vs. 68.6, 73 vs. 63.9; p=0.032-0.045). For alloys, 37.5% [CI: 0.32; 0.43] used 110 µm and 0.2 MPa. Instruction/training, an age of 30 or above, an experience of 10 years or more or being a dental technician were associated with a higher number of participants than was expected choosing this surface pretreatment (42 vs. 33.7, 78 vs. 69.3, 79 vs. 64.5, 59 vs. 35.6; p<0.001–0.043). Furthermore, daily APA was associated with a higher number of participants (70 vs. 53.4) using these APA parameters than observed for participants who perform airborne particle abrasion weekly (25 vs. 31.9; p<0.001).

**Fig 6:**
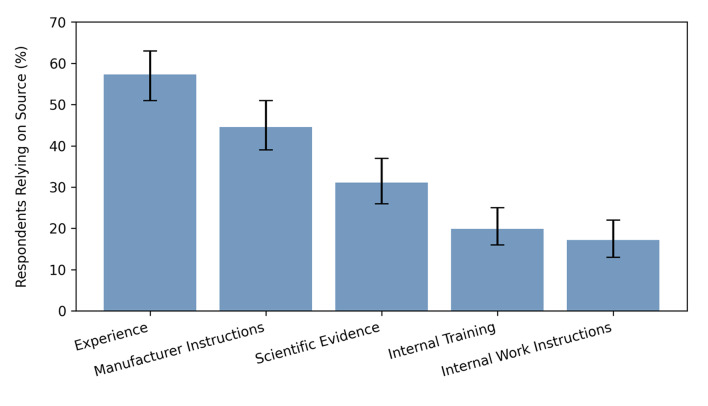
Percentages of specifications for the airborne particle abrasion parameters (with 95% CI).

For APA of zirconia, polymer-based restorations, or alloys, respectively 19.9% [CI: 0.16; 0.25], 26.2% [CI: 0.21; 0.32] and 24.7% [CI: 0.20; 0.30] of the participants indicated that these parameters were unclear to them. Just above half of the participants (149/267 [CI: 0.50; 0.62]) employed a distance of 5–10 mm between the restoration and the APA nozzle, using an angle of 45° [CI: 0.50; 0.62]. 23.6% [CI: 0.19; 0.29] did not consider the angle when using APA. The majority of the participants (57.3% [CI: 0.51; 0.63]) based the APA parameters they used on their experience, followed by the manufacturers’ instructions (44.6% [0.39; 0.51]) and scientific evidence (31.1% [CI: 0.26; 0.37]). 19.9% [CI: 0.16; 0.25] of the participants indicated internal training and 17.2% [CI: 0.13; 0.22] internal work instructions as the specifications for the used APA parameters (Fig 6).

Less than half of the participants (40.8% [CI: 0.35; 0.47]) could imagine using APA for silicate-based ceramics instead of etching them. A high majority of participants (95.1% [CI: 0.92; 0.97]) indicated using APA on a polymer-based crown for less than 20 s in comparison with airborne particle abrading for more than 60 s. Being a trainee/student or dental assistant with less than 10 years of experience was associated with a higher number of participants experiencing APA for more than 60 s than expected (5 vs 1.4, 4 vs 0.9, 11 vs 4.8; P <0.001–0.002).

**Fig 7:**
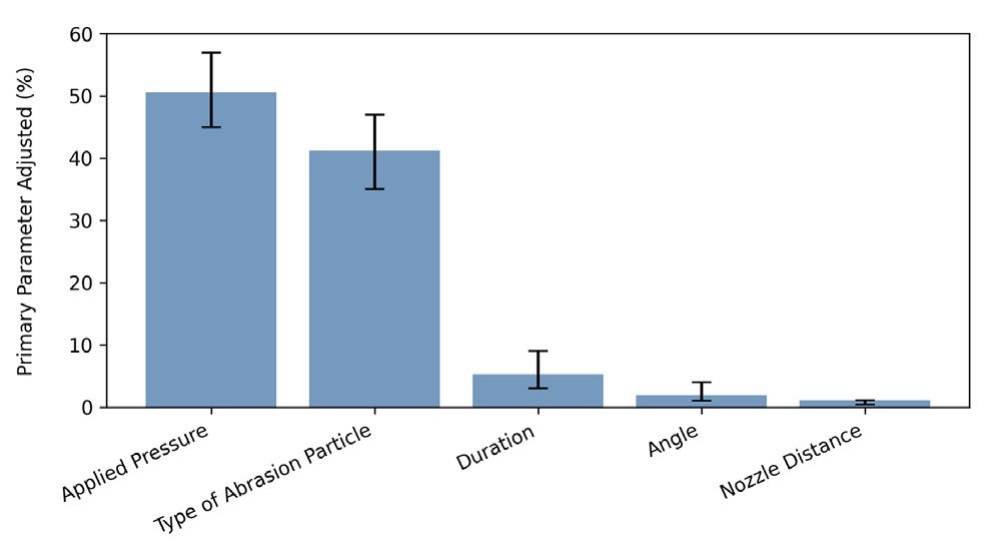
Percentages of the primary parameter adjusted by the participants (with 95% CI).

The applied pressure was the primary parameter adjusted material-specifically at the APA unit (50.6% [CI: 0.45; 0.57]), followed by the type of abrasion particle (41.2% [CI: 0.35; 0.47]), APA duration (5.2% [CI: 0.03; 0.09]), angle between nozzle and restoration (1.9% [CI: 0.01; 0.04]) and distance between nozzle and restoration (1.1% [CI: 0.004; 0.01]) (Fig 7).

Participants showed a variety of cleaning protocols, reporting the use of water steaming (36.3% [CI: 0.31; 0.42]), compressed air (29.2% [CI: 0.24; 0.35]), ultrasonic bath (25.1% [CI: 0.20; 0.31]), disinfection (6.7% [CI: 0.04; 0.10]) or no additional cleaning (2.6% [CI: 0.01; 0.05]). Surfaces were airborne particle abraded for enlarging the surface (84.6% [CI: 0.80; 0.88]), cleaning (78.3% [CI: 0.73; 0.83]), increasing the wettability (74.9% [CI: 0.69; 0.80]), divesting (44.9% [CI: 0.39; 0.51]) and increasing the stability of the restoration (24% [CI: 0.19; 0.29]).

### Operating Procedures for the APA Unit

Most of the participants (67.4% [CI: 0.62; 0.73]) indicated that they perform APA when standing, with 32.6% [CI: 0.27; 0.38] working in a sitting position. Being a dental assistant or dentist was associated with performing APA while sitting (13 vs 5.5 and 50 vs 41.4), while being a dental technician was associated with performing APA when standing (79 vs 64.7; P<0.001). 64% [CI: 0.58; 0.70] of the participants used gloves when airborne particle abrading, with dental assistants or dentists being associated with using gloves in a higher number (15 vs 10.9 and 86 vs 81.3; P = 0.030). Most participants cleaned their APA unit regularly (77.5% [CI: 0.72; 0.82]), specifying a moderate (61.4% [CI: 0.55; 0.67]) or low cleaning effort (33.3% [CI: 0.28; 0.84]). Cleaning was performed by suction (73% [CI: 0.67; 0.78]), wiping (25.8% [CI: 0.21; 0.31]) and/or using disinfectant wipes (10.9% [CI: 0.08; 0.15]).

### Pretreatments For Adhesive Placement of Indirect/Semi-Direct Dental Restorations

**Fig 8:**
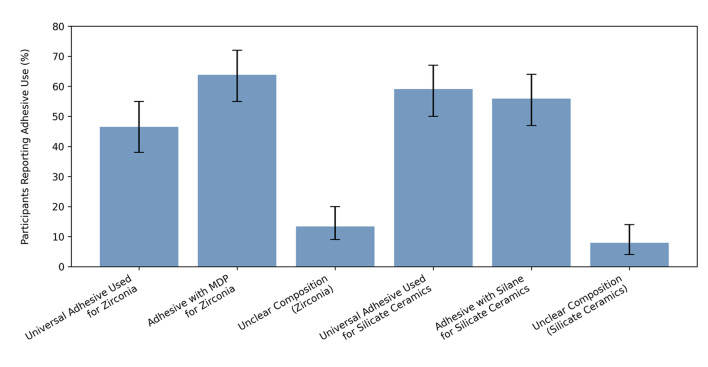
Use of adhesives for zirconia vs silicate-based ceramics (with 95% CI).

For the following questions concerning the fixation of dental restorations, solely the answers of the dentists were analyzed, as this topic falls into the responsibility of this profession. When asked how to lute zirconia crowns, which were temporarily cemented with eugenol-containing cement, 85.8% [CI: 0.79; 0.91] indicated using conventional non-adhesive cementation. Most participants did neither clean silicate-based ceramic (84.3% [CI: 0.77; 0.90]), which had been etched with hydrofluoric acid, nor clean airborne particle-abraded zirconia (96.9% [CI: 0.92; 0.99]) crowns using phosphoric acid. 24.4% [CI: 0.18; 0.33] used special cleaning products after surface pretreatment. Universal adhesives were employed for zirconia by 46.5% [CI: 0.38; 0.55] and for silicate-based ceramic restorations by 59.1% [CI: 0.50; 0.67] of the participants. 63.8% [CI: 0.55; 0.72] of the participants used an adhesive with MDP monomers for luting zirconia. For silicate-based ceramic, 55.9% [CI: 0.47; 0.64] employed an adhesive containing silane. For both questions, respectively 13.4% [CI: 0.09; 0.20] and 7.9% [CI: 0.04; 0.14] of the participants indicated that the composition of the adhesive system was unclear to them (Fig 8).

Just over one-third of the participants (37.8% [CI: 0.30; 0.46]) specified that they would like to avoid hydrofluoric acid etching. When asked how to treat an already alumina airborne particle abraded restoration after trying-in, answers were split between a repeated APA (48.0% [CI: 0.40; 0.57]) and no repeated APA (52.0% [CI: 0.43; 0.60]). For debonded restorations, a high majority (94.5% [CI: 0.89; 0.97]) perform repeated APA. For both scenarios, performing repeated APA was dependent on access to an APA unit (p<0.001-0.009). A small number of the participants (12.4% [CI: 0.09; 0.17]) experienced a restoration no longer being insertable following APA. Three out of four participants (75.7% [CI: 0.70; 0.80]) would like to have more information about APA as a part of their training, studies or further education.

### Expert Consensus

Based on the expert consensus, the level of agreement on each recommendation was determined and varied between 52% and 100%, with 21/23 recommendations showing an average agreement level of 93%. For 15 out of the 23 recommendations, the level of agreement exceeded 90%. For the three recommendations, the level of agreement was above 80% and 70%. Two recommendations showed a level of agreement of about 50%.

## DISCUSSION

The aim of this investigation was to analyze the handling and use of APA and other pretreatments for adhesive placement of dental restorations in German-speaking countries. The tested null-hypotheses stating that neither the participants’ sex, age, experience, activity, profession nor their previous contact with airborne particle abrasion (access to APA unit, prior experience with APA, frequency of APA, instruction/training) have an impact on their use of APA or workflow for bonding a dental restoration were rejected.

Airborne particle abrasion is an indispensable tool in the luting of restorations in modern dentistry, but the full utilization of this potential depends on the equipment available in dental facilities. In this survey, access to an APA unit was associated with a higher number of participants performing alumina APA prior to the luting of a restoration. With multiple studies showing this to be a vital step for surface pretreatment,^9,62,87 APA^ units should be accessible for pretreatment of dental restorations prior to luting to ensure long-term success.

**Fig 9:**
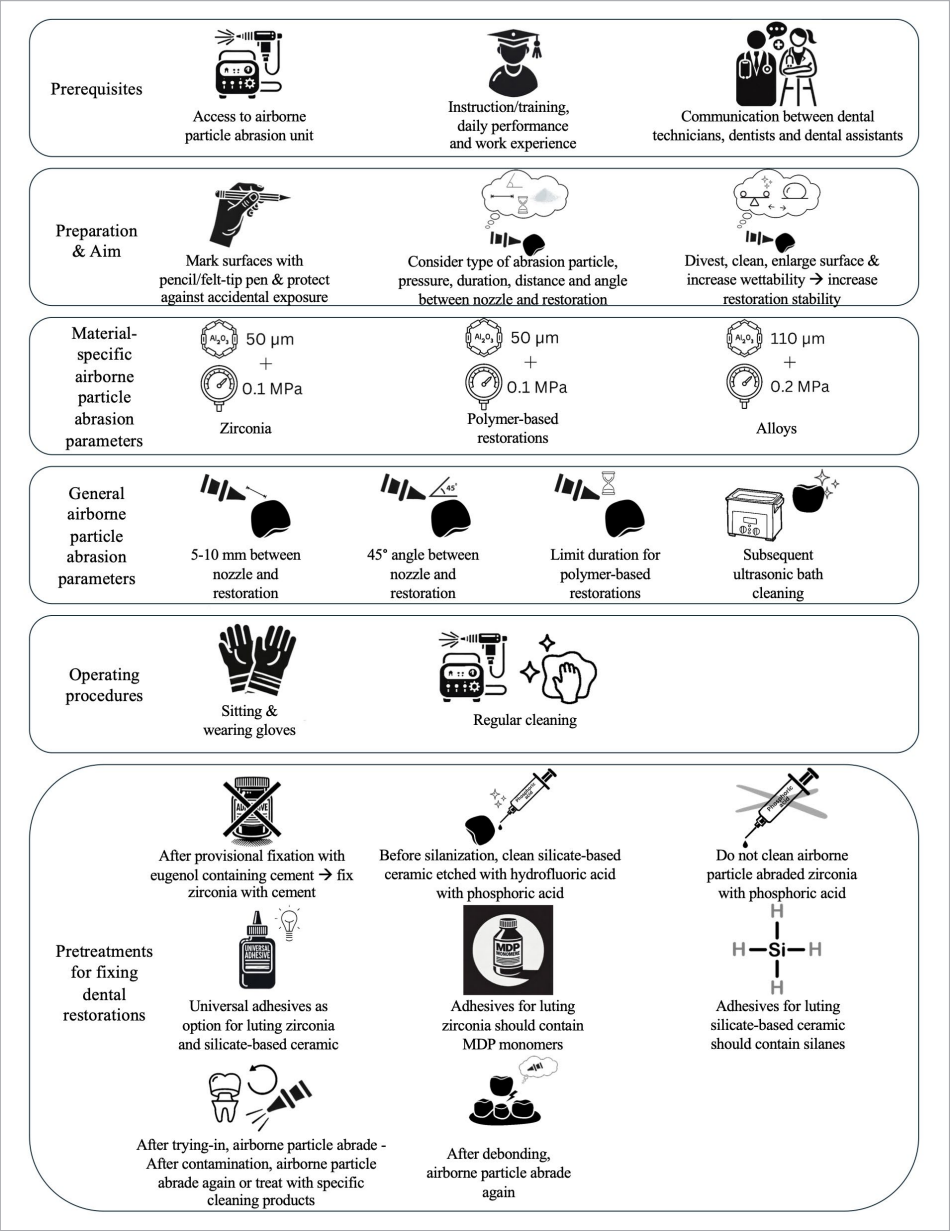
Graphical illustration of the recommended adhesive luting workflows of zirconia, alloy, and polymer-based restorations.

Notably, two-thirds of the participants had not received instructions or training on alumina APA. The importance and complexity of correctly performing this task for a multitude of materials highlight the need for regular, targeted training of dental professionals. Experienced dental technicians and dentists could act as mentors by passing on their knowledge and skills in the daily workflow. Accessible, clear, and detailed work instructions, eg, printing out an overview of the different APA parameters for the materials in use in a specific setting (Fig 9) or using pre-programmed units that automatically adjust the APA parameters when choosing a restorative material, can help inexperienced colleagues and team members. With scientifically validated choices for APA parameters being associated with an age of ≥30 years, an experience of ≥ 10 years, as well as prior instruction/training and using APA daily, users should be instructed and trained on performing alumina APA. If feasible, APA should furthermore be performed by users who perform APA daily and possess extensive work experience.

The survey results reveal an interesting distribution of tasks between the different dental professions in relation to the performance of APA. The different percentages regarding the responses of dentists and dental technicians to the question of who performs APA suggest that there is a lack of either clear agreement or established protocols for the workflow, including the communication between the lab technician and the dental practitioner. This situation is amplified by just over half of the participants communicating about APA in their working environment. Consequently, restorative materials may be airborne particle abraded several times or not at all, entailing an unnecessary weakening of the restoration caused by repeated airborne particle abrasion^11^ or insufficient surface properties regarding bonding.^45,62,75^ To ensure a sufficient treatment of all surface areas of the restoration, a prior marking with a pencil or felt-tip pen is advised. At the same time, surfaces that should not be airborne particle abraded, eg, connectors of multi-unit fixed dental prostheses (FDPs), should be protected against accidental exposure to APA to prevent rough surfaces which might cause increased plaque accumulation. With previous research showing varying results in dependence of the applied abrasion parameters,^38^ the type of abrasion particle, the applied pressure, the APA duration, the distance between the nozzle and restoration and the angle between the nozzle and restoration should all be considered when using APA.

Nearly half of the participants reported alumina airborne particle abrading zirconia with 50 µm and 0.1 MPa. According to the literature, both overly aggressive and insufficient APA can have detrimental effects. Excessive APA pressure (>0.2 MPa) can damage the zirconia surface, leading to microcracks and a weakening of the material.^91^ On the other hand, if the applied pressure is too low, the necessary surface roughness for proper bonding may not be achieved, which can result in inadequate adhesion and long-term failure of the restoration.^26,92^ Using 50 µm and 0.1 MPa has been considered adequate to ensure a durable bond to zirconia on, eg, titanium abutments.^24^ A systematic review did not report an impact of the particle size on long-term bond strength.^39^ Just about a third of the participants used the same parameters to abrade polymer-based restorations with alumina airborne particles. With this material group encompassing a wide variety of subgroups, such as filled composite resins, PMMA-based materials, and PEEK, the literature diverges on the ideal APA parameters for this group.^3,21,23,27,29,32,37,38,63,66,68,71,75,81^ Following the manufacturers’ recommendations, some polymer-based restorations are only allowed to be etched.

In addition, the composition of the employed adhesive and luting material has to be considered, as for some adhesives, APA with silicon oxide-coated alumina particles may be preferable.^75^ These challenges are reflected in the fact that 9% of the experts are uncertain regarding recommendation 7 (Table 3), which proposes using 50 µm and 0.1 MPa alumina APA for polymer-based restorations. For PEEK, a systematic review reported an increase in shear bond strength by applying APA with 50 µm alumina particles.^23^ As discussed for zirconia, the potential detrimental effects of extensive APA for polymer-based restorations have to be considered, with extended APA causing surface damage, leading to microcracks or an overly rough surface that can weaken the polymer material and reduce its overall flexural strength.^16^ When asked about APA durations, nearly all the participants indicated using APA on a polymer-based crown for less than 20 s in comparison with airborne particle abrading for more than 60 s, showcasing a negative previous experience or knowledge about the dangers of overexposing polymer-based restorations to APA.^31^ For alloys, 37.5% of the participants reported using alumina APA with 110 µm particles and a pressure of 0.2 MPa, with previous research showing a positive effect of a bigger particle size and higher pressure on the shear bond strength.^14,22^ In this context, it is important to highlight that employing lower pressures or smaller particle sizes could lead to inadequate bonding.^35^ In conclusion, it must be considered that ceramics are brittle materials that may suffer from excessive APA. For ductile and softer polymer-based materials, a potential over-exposure to APA may result in insufficient restoration margins. With alloys being both ductile and at the same time harder, they are less prone to abrasion. The fact that 20–25% of the participants openly stated that the APA parameters for these materials were unclear to them emphasizes the need for further research and education on this subject.

Almost a third of the participants indicated that they airborne-particle abraded silicate-based ceramics with alumina. Slightly more than 40% stated this for lithium-disilicate-glass ceramics. Current literature recommends that silicate-based ceramics should be etched with hydrofluoric acid for a specific time (20–60 s) prior to adhesive luting, depending on the silicate percentage, in order to establish a sufficient bond to the luting composite through the resulting etching pattern.^19,72^ The finding that 37.8% of participants indicated a desire to avoid hydrofluoric acid etching highlights the awareness among professionals of the dangers associated with this pretreatment, underscoring the need for innovative alternative pretreatment methods for silicate-based ceramics. Nonetheless, hydrofluoric acid (HF) etching can be advantageous in many cases and remains the recommended and gold-standard surface pretreatment for etchable ceramics prior to adhesive luting, as it is often easier, faster. APA requires a dedicated device, time-consuming setup, and carries occupational risks such as dust inhalation. Moreover, specific composite CAD/CAM restorations containing glass fillers can also be etched with HF, achieving comparable bonding effectiveness to APA. HF etching does not require a specialized device and can thus be preferable in certain clinical situations.

The latest studies indicate that the dangerous acid can be dispensed with when luting silicate-based restorations if the restorations are alternatively being airborne particle abraded using 25 µm or 50 µm alumina particles at a pressure of 0.1 MPa.^12,36^ It is therefore conceivable that practitioners will prospectively not only airborne particle abrade silicate-based ceramics to de-embed pressed silicate-based ceramics and effectively and quickly remove the remains of the embedding material,^52,73,74^ but also use this surface treatment to enhance bonding and thus replace hydrofluoric acid etching.

If feasible, a distance of 5–10 mm should be maintained between the APA nozzle and the restoration when performing alumina APA to achieve a homogenous abrasion surface. In addition, an angle of 45° should be used between the APA nozzle and the restoration when performing alumina APA, as the literature has shown this to result in a more even and homogenous distribution of surface defects.^18,64^ Consistently using a specific angle during APA is, however, often unfeasible, especially for slim and long hollow spaces. This challenge is underlined by 9% of the experts being uncertain with regard to the corresponding recommendation 10 (Table 3).

A multitude of protocols are in use following alumina APA, ranging from no additional cleaning, to the use of compressed air or water steaming, to cleaning in an ultrasonic bath. ^5,76^ With higher bond strengths being observed following ultrasonic cleaning (7), it is recommended that specimens should be cleaned in an ultrasonic bath after performing alumina APA, using 99% isopropanol for 3 min, preferably. The benefits of APA for divesting, cleaning, enlarging the surface, or increasing the wettability and thus the stability of the restoration have been shown in an investigation.^18^ In consequence, dental technicians perform various tasks that require varying degrees of precision. For example, when devesting cast models, which calls for less technical finesse than the APA of dental crowns, dental technicians tend to use the abrasion equipment while standing. This enables fast and efficient workflows. With a sitting position presumably allowing for a higher precision in the handling of the unit, it is nonetheless recommended that APA units should be operated when sitting, especially when working on definitive prosthetic restorations. The results of the survey showing dental assistants or dentists being associated with a sitting position may indicate that this factor is already under consideration.

The data on the use of gloves during APA shows that 64% of participants wear gloves. Dentists and dental assistants tended to use gloves more frequently when performing this activity, which could indicate a higher awareness of occupational safety in these groups, paired with a higher frequency of glove interaction during their routine working day. In many cases, the less frequent use of gloves by dental technicians could be due to less stringent safety protocols or a lower perception of risk. It is important that occupational health and safety guidelines are consistently applied to all occupational groups to ensure uniform safety standards, calling for the wearing of gloves when operating APA units. Cleaning protocols of the APA unit varied between participants, with the use of suction being the most common. If available, recommendations provided by the APA unit manufacturer should be adhered to. Regular cleaning and inspection are recommended to ensure that, eg, a clogged tube does not entail a reduced pressure and that the chosen APA parameters are in fact being successfully executed.

The results of this survey on surface treatment and adhesive luting preferences for dental restorations highlight important trends, while indicating areas where more education may be needed. Knowledge about the potential negative interaction between an eugenol-containing cement and resin-based materials^34^ seems to be widespread, with over 80% of dentists indicating that they would employ non-adhesive cementation in a setting where an eugenol-containing cement had been used. However, the issue of polymerization inhibition by eugenol may be overstated. During tooth pretreatment by etching with phosphoric acid or by polishing with pastes to remove adherent temporary cement, eugenol residues could be sufficiently removed so that standard procedures for adhesive luting procedures are possible without impairing bond strength. In addition, it should be noted that simple APA alone is sufficient to eliminate the polymerization-inhibiting effects of eugenol-containing temporary cements, thereby allowing reliable adhesive luting protocols even after provisional restorations. Thirty-five percent of the experts did, however, not agree with the corresponding recommendation 16 (Table 3). This may be because the bond strength that is reached using adhesive luting in this setup still surpasses that achieved by using a cement and a meta-analysis pointing out that after 14 days of placement, eugenol-based restorations did not impact the bonding of adhesives to dentin.^15^ It should also be noted that conventional cementation typically requires a macro-retentive preparation design, whereas full adhesive luting protocols can be used even with non-retentive preparations by relying on micromechanical and chemical bonding. Furthermore, a pretreatment of the bonding surface can successfully remove residual eugenol.^10^ The focus should thus be on taking the ingredients of provisional fixation materials into account before initiating the definitive fixation procedure and considering these during the pretreatment of the bonding surfaces.

While research has shown benefits of cleaning silicate-based ceramics following hydrofluoric acid etching using phosphoric acid,^50^ this is not implemented in daily practice. This may be due to the increased time and effort that this cleaning procedure calls for, with each additional work step increasing the risk of operator errors further, and is reflected by 35% of the experts disagreeing with the corresponding recommendation 17 (Table 3). With 96.9% of participants indicating that they did not clean airborne particle-abraded zirconia using phosphoric acid, the danger of using this cleaning method and thus prematurely occupying future binding sites^76^ seems to be limited. Only one-fourth of the participants used cleaning products after surface pretreatment. One of the first investigations examining the potential of an MDP-containing cleaner to remove provisional cement residues reported similar tensile bond strength values following treatment with the cleaner in comparison with polishing.^77^ Regarding the cleaning of a zirconia restoration that had been contaminated with saliva or blood, a pretreatment with ZirClean, Ivoclean or Katana Cleaner led to comparable values as observed for non-contaminated specimens.^8,76,79^ Thus, cleaning products may represent a valuable addition for treating contaminated surfaces.

The data shows a relatively widespread use of universal adhesives, with more participants using universal adhesives for silicate-based ceramics than for zirconia. When comparing the shear bond strength of universal adhesives to human enamel and dentin with two-step adhesives, 3/5th of the universal adhesives showed comparable values to enamel, and 4/5th comparable or higher values to dentin,^25^ corroborating a high potential for bonding to the natural tooth structure. To ensure a high bond strength to lithium-disilicate-glass-ceramics, universal adhesives should contain silane, with research highlighting that using a separate primer containing silane and phosphate monomer can provide a more durable bond than silanes incorporated in universal adhesives.^17,60^ A systematic review concluded that the bond strength to lithium-disilicate-glass-ceramics is higher when using hydrofluoric acid etching and a silicate-ceramic primer than reported for employing hydrofluoric acid and silane-containing universal adhesives.^40^ The latest investigations do, however, show promising results for the latest universal adhesive product available on the market.^6,78^ The bonding efficacy of universal adhesives to zirconia substrates displayed similar bond strength values when compared with phosphate-silane- and phosphate-based primers.^42^ Nonetheless, it must be emphasized that “dedicated restoration primers,” which primarily contain silane and 10-MDP dissolved in a solvent, generally exhibit significantly higher bonding effectiveness than universal adhesives containing the same components in an aqueous acidic environment. Research indicates that silane is unstable in acidic, water-based solutions, and the presence of multiple monomers in universal adhesives may interfere with the ability of 10-MDP and silane to adequately interact with the restoration surface. Therefore, recommending a separate “restoration primer” for the restorative material – in addition to a “tooth primer” (eg, an adhesive) for the tooth substrate – remains clinically prudent for predictable and durable results.

In conclusion, universal adhesives may represent a less technique-sensitive option for successfully luting silicate-based ceramic and zirconia restorations.^82,84^ Seventeen percent of the experts disagreeing with the corresponding recommendation 19 (Table 3) does, however, highlight the need for additional research in this field. Remarkably, there is considerable uncertainty among participants regarding the composition of adhesives, with only 63.8% and 55.9% of participants using adhesives with MDP monomers for luting zirconia or adhesives with silane for luting silicate-based ceramics, respectively, whereas the literature conclusively shows that adhesives for luting a zirconia restoration should contain MDP monomers,^2^ while adhesives for silicate-based ceramic restorations should include silanes.^69^ With these products having been established on the dental market for more than 20 years, these findings still suggest a lack of education and information in this area. Previous surveys from 2007, 2011, and 2015 have shown a range of 36–69% of participants using evidence-based fixation protocols for silicate-based ceramics, with 7–14% of participants employing evidence-based protocols for zirconia.^33^ In a follow-up survey from 2019, evidence-based treatments for silicate-based ceramics declined to 38%, while knowledge about zirconia increased to 62%.^56^ When comparing these results to the present survey, knowledge about silicate-based ceramics seems to have once again improved, with the data on zirconia staying constant.

The results on airborne particle abrading restorations after trying-in that have already been airborne particle abraded with alumina and how to treat debonded restorations raise interesting questions about the accessibility of APA units in dental practices. Participants were almost evenly divided on whether or not to re-abrade with alumina after the try-in of a restoration (48% for, 52% against a repeated pre-treatment). With the literature showing a negative impact of contamination with saliva or blood on the bond strength,^56^ a repeated APA promises a clean surface. In this context, the newly introduced cleaning products may represent a valid alternative, as these do not threaten to weaken the restoration with repeated APA, while ensuring a clean surface after contamination.^79^ If an APA unit is available in the dental practice, practitioners should communicate with the laboratory and only perform APA after the try-in, thereby avoiding unnecessary duplication of steps.. In contrast to the situation after try-in, a high majority of the participants believe that re-abrasion is necessary for debonded restorations. This reflects a clear consensus and shows the crucial role of APA in removing surplus luting material and enabling the formation of a novel, adequate bond.^61^ In both scenarios, the performance of APA, of course, depends significantly on the potential access to an APA device. Lack of access to such a device will thus compromise the quality of treatment, especially in less well-equipped practices. In the long run, an inadequate bond may result in the formation of secondary caries, a weakening of the restoration and the long-term failure of the treatment, emphasizing the importance of adequate surface pretreatments and luting protocols.^48^

The expert consensus reaching an average agreement level of 93% for 21/23 recommendations shows a high conformity on the part of the experts with the proposed recommendations.

### Limitations

In this survey, the term “resin” was used, with no specific distinction being made between PMMA and composite resins. This could influence the interpretation of the results, as the two material groups have different properties and applications. Additionally, the composition of composite resin materials varies strongly among available products. Similarly, the term “zirconia” was employed, encompassing all generations of zirconia without further differentiation.^80^ With some recommendations, notably recommendations 4, 13, 14, and 19 (Table 3), summarizing different aspects within one recommendation, experts may have chosen that they were uncertain or disagreed with the recommendation, despite supporting individual aspects of the proposed APA parameters and other pretreatments. Unfortunately, in the present survey, the size of the APA powder was not clearly separated from the pressure in the respective questions. Similarly, regarding alumina particle size, it would have been preferable to differentiate between “<50 µm” and “>50 µm” to allow more precise data collection and analysis. This limitation may affect the ability to draw definitive conclusions from the survey results. Furthermore, the survey did not consider APA with silicon-oxide-coated alumina particles as a surface treatment, which is, however, a common practice in restorative treatment.^13,58^ Future studies are necessary to investigate the use of silicon oxide coating and its role in the dental field. It is critical to note that the survey was specifically sent to universities, which may result in an overrepresentation of participants from the academic field, while practitioners in private practices may hold differing views and strategies. Participation was voluntary and took approximately 7 min, which may have resulted in the self-selection of motivated participants. Additionally, the survey included trick questions to assess participants’ prior knowledge and expertise. While yielding important insights, this may have discouraged knowledgeable participants from completing the survey.

### Clinical Relevance

This research highlights how critical access to APA devices is for successful restorative dental treatments. The results indicate that both the availability of this technology and targeted training of all dental professionals significantly influence clinical decision-making and thus may impact long-term outcomes. Therefore, all dental facilities, regardless of size or location, should be adequately equipped with APA technology and offer appropriate training to ensure a high quality of care.
